# The convergence of racial and income disparities in health insurance coverage in the United States

**DOI:** 10.1186/s12939-021-01436-z

**Published:** 2021-04-07

**Authors:** De-Chih Lee, Hailun Liang, Leiyu Shi

**Affiliations:** 1 Department of Information Management, Da-Yeh University, No.168, University Rd., Dacun, Changhua, 51591 Taiwan; 2 School of Public Administration and Policy, Renmin University of China, No.59 Zhongguancun, Beijing, 100872 China; 3 Johns Hopkins Primary Care Policy Center, 624 N. Broadway, Baltimore, MD 21205 USA; 4 Johns Hopkins Bloomberg School of Public Health, 624 N. Broadway, Baltimore, MD 21205 USA

**Keywords:** Race, Ethnicity, Disparity, Health insurance

## Abstract

**Objective:**

This study applied the vulnerability framework and examined the combined effect of race and income on health insurance coverage in the US.

**Data source:**

The household component of the US Medical Expenditure Panel Survey (MEPS-HC) of 2017 was used for the study.

**Study design:**

Logistic regression models were used to estimate the associations between insurance coverage status and vulnerability measure, comparing insured with uninsured or insured for part of the year, insured for part of the year only, and uninsured only, respectively.

**Data collection/extraction methods:**

We constructed a vulnerability measure that reflects the convergence of predisposing (race/ethnicity), enabling (income), and need (self-perceived health status) attributes of risk.

**Principal findings:**

While income was a significant predictor of health insurance coverage (a difference of 6.1–7.2% between high- and low-income Americans), race/ethnicity was independently associated with lack of insurance. The combined effect of income and race on insurance coverage was devastating as low-income minorities with bad health had 68% less odds of being insured than high-income Whites with good health.

**Conclusion:**

Results of the study could assist policymakers in targeting limited resources on subpopulations likely most in need of assistance for insurance coverage. Policymakers should target insurance coverage for the most vulnerable subpopulation, i.e., those who have low income and poor health *as well as* are racial/ethnic minorities.

## Background

It is well-known that in the United States there are significant racial and income disparities in health insurance coverage. Studies conducted within the past 5 years have examined the relationship between social structure variables (such as race, ethnicity and income) and insurance coverage to suggest disparities between certain subgroups. In 2018, non-Hispanic Whites had the highest health insurance coverage at 94.6%, followed by Asians at 93.2%, Blacks at 90.3%, and Hispanics at 82.2%, among population aged 18 and over [1]. Similarly, another study compared insurance coverage rate before and after the Medicaid expansion in 2015 across racial/ethnic groups and found that the uninsured rate only changed slightly in minority groups [2]. There was only a one percentage point decrease in uninsured gap between Hispanic and non-Hispanic Whites in 2015 and the uninsured rate was three times higher between the two subpopulations. Likewise, lower household income was associated with lower health insurance coverage rate [1, 3, 4]. People in households with an annual income of $150,000 or more had a higher percentage of insurance coverage (96.8%) than people in households with income of less than $25,000 (86.2%). Another method to assess income level is income-to-poverty ratio. People in higher income-to-poverty ratio groups had higher health insurance coverage rates in general. People living at or above 400% of poverty had higher coverage (96.6%) than people living below 100% of poverty (83.7%).

However, little is known about the combined effect of race and income on insurance coverage. For example, does racial/ethnic minority status have an independent effect on health insurance coverage regardless of income level implying systemic racial disparity in insurance coverage? Or is lack of insurance primarily associated with low income? Since racial/ethnic minorities are more likely to have lower income than Whites, could racial disparity in insurance coverage mainly reflect racial disparity in income distribution? Sorting out the respective influences of race and income on insurance coverage requires a new conceptual and analytic framework that examines the effect of the *convergence* of risk factors rather than addressing them individually or independently. Since health insurance contributes significantly to ensuring access to, and continuity of, care [5–7], and continuity of care affects quality and health outcomes [8–10], ensuring health insurance coverage is essential in improving health status. As demographic shifts and socioeconomic trends in the US will result in minority populations becoming the majority within the twenty-first century, fundamental improvement of the nation’s health cannot be accomplished without corresponding improvement in the health of American minorities.

This study applies the “general framework to study vulnerable populations” and examines the combined effect of race and income on health insurance coverage in the US. Compared with previous studies using the vulnerability concept to evaluate the quality of primary care experienced by health center patients [11–13], our study provided additional contribution to the literature by advancing the knowledge of the combined effect of race and income on health insurance coverage which could assist policymakers in targeting limited resources on subpopulations likely most in need of assistance for insurance coverage. Having health insurance is also crucial during pandemics like COVID-19 as the insured are more likely to get help in triaging, testing, treatment, and recovery. Those without health insurance could face systemic barriers accessing COVID-19 testing and treatment services. Racial/ethnic and income disparities in health and healthcare persist and even worsen over time despite national and local policies and programs aimed at their reduction or elimination. These disparities are further exacerbated under COVID when racial/ethnic minorities and low-income individuals are more likely to be susceptible to infection and mortality and less likely to get timely treatment and vaccination [14–16]. These persistent and worsening disparities call for a new framework to understand these disparities and new approach to address them. Heretofore, racial/ethnic and income disparities are largely understood as separate disparities in the literature and their interactions often neglected in both policies and programs. Our vulnerability framework allows the simultaneous consideration of predisposing (such as race/ethnicity), enabling (such as income), and need (such as health status) factors in their influence on healthcare, thus filling a critical gap in the literature and affording policymakers opportunity to incorporate multiple risk (vulnerability) factors in their policies and programs.

## Methods

### Data

Data for this analysis came from the Household Component (HC) of the 2017 Medical Expenditure Panel Survey (MEPS), a nationally representative survey of the civilian noninstitutionalized population of the United States, that was publicly available for download (https://www.meps.ahrq.gov/mepsweb/data_stats/download_data_files_detail.jsp?cboPufNumber=HC-201). All statistical analyses accounted for the complex sampling design, and included all the participants who had a positive sampling weight (PERWT17F). For analyses, we further restricted the sample to those under 65 years old at the time of being surveyed. Since people aged over 65 (except for those who did not meet the criterion of having minimum 40 quarters of work-related payment contributions or were undocumented or otherwise ‘illegal’ residents the US) are eligible for government Medicare insurance regardless of income, their inclusion could confound the effect of income on insurance coverage.

### Outcome

The primary outcome of the study was insurance coverage status, a categorical variable with five levels: (1) publicly insured: having public insurance for 12 consecutive months in 2017; (2) privately insured: having private insurance for 12 consecutive months in 2017; (3) dually insured: having public or private insurance for 12 consecutive months in 2017 AND for at least one of these months the participant had both public and private insurance coverage, including the scenario that for 1 month the participant had private insurance and for another month the participant had public insurance; (4) insured for part of the year: having either public or private insurance for at least 1 month but less than 12 months of 2017; and (5) uninsured: having no insurance coverage for 12 consecutive months in 2017. Including the insured for part of the year allows us to look at health insurance as a continuum from those fully insured, to insured for part of the year, and then to the uninsured. Results can then be interpreted more precisely and the gradient effect of insurance can then be looked at. Including this group could also enhance the sample size and hence power for the analysis. On the other hand, lumping this group with the insured or uninsured could dilute the effect of insurance.

We used three sets of variables to construct the insurance coverage status variable – any insurance in a month (INSJA17X – INSDE17X), any public insurance in a month (PUBJA17X – PUBDE17X), and private insurance in a month (PRIJA17 – PRIDE17). Public insurance referred to TRICARE, Medicare, Medicaid or State Children’s Health Insurance Program (SCHIP), or other public hospital/physician programs. Private insurance included union or employer group insurance, non-group insurance, other group insurance, and private insurance through federally facilitated, state-based or state partnership marketplace or exchange. We considered a subject as having any insurance if covered by any of these insurance sources above.

### Exposures

Operationalization of the exposures used for this study was based on the “general framework to study vulnerable populations” promulgated by Shi and Stevens [17]. According to Shi and Stevens, vulnerability refers to the likelihood of experiencing poor health and is determined by a convergence of predisposing, enabling, and need characteristics. In their access to care framework [18, 19], Aday and Andersen described predisposing, enabling, and need characteristics respectively as follows. Predisposing characteristics was described as those that describe the propensity of individuals to use health services including basic demographic characteristics (e.g., age, sex, and family size), social structure variables (e.g., race and ethnicity, education, employment status, and occupation), and beliefs (e.g., general beliefs and attitudes about the value of health services). Enabling characteristics was described as the means individuals have available to them for the use of services including resources specific to individuals and families (e.g., income and insurance coverage) and attributes of the community or region in which an individual lives. Need characteristics was described as health status or illness, which is the most important cause of health services use. Thus, individuals are most vulnerable if they experience a convergence of predisposing, enabling, and need attributes of risk.

Based on this framework, we identified measures within MEPS that denote predisposing, enabling, and need attributes of risk. We combined three of these variables into a new vulnerability measure that reflects the convergence of predisposing, enabling, and need attributes of risk. These were race (predisposing dimension), income (enabling dimension), and self-perceived health status (need dimension), and are among the most, although not the only, significant indicators of vulnerability. It is possible to create a measure incorporating other vulnerable attributes (e.g., the more objective chronic illness measure for health status, the behavioral risks such as smoking, alcohol, and drug abuse for predisposing factor). However, the trade-off is that the resulting sample size for some subgroups would be too small for comparative analysis (e.g., chronic illness is likely to be more concentrated among the elderly population). To avoid small subgroup sample size, we further re-coded these variables into dichotomous categories so that our final vulnerability measure was limited to eight categories: (1) the minority-low-income-bad health group (the most vulnerable group with vulnerable attributes in all three dimensions), (2) the minority-low-income-good health group, (3) the white-low-income-bad health group, (4) the white-low-income-good health group, (5) the minority-high-income-bad health group, (6) the minority-high-income-good health group, (7) the white-high-income-bad health group, and (8) the white-high-income-good health group (this is the least vulnerable group with none of the three vulnerable attributes measured).

Minority included all non-white racial and ethnic groups including blacks, Hispanics, Asians, American Indians, and others. Measure of income was based on the variable family income as a percentage of poverty within MEPS (POVCAT). Family income comprised annual earnings from wages, salaries, bonuses, tips, commissions; business and farm gains and losses; unemployment and workman’s compensation; interest and dividends; alimony, child support, and other private cash transfers; private pensions, IRA withdrawals, social security, and veterans payments; supplemental security income and cash welfare payments from public assistance, aid to families with dependent children, and aid to dependent children; gains and losses from estates, trusts, partnerships, S corporations, rent, and royalties; and a small amount of “other” income. Family income excluded tax refunds and capital gains. Person-level income totals were then summed over family members to yield the family-level total. POVCAT was constructed by dividing family income by the applicable poverty line (based on family size and composition), with the resulting percentages grouped into five categories: negative or poor, near poor, low income, middle income, and high income. For the purpose of this study, we grouped ‘negative or poor, near poor, low income’ as ‘low income’ (less than or equal to 299% federal poverty line) and ‘middle income, high income’ as ‘high income’(300% or more than federal poverty line). For health, ‘good health’ included excellent, very good, and good health, whereas ‘bad health’ included fair and poor health. Self-rated health has strong predictive validity for mortality, morbidity, and mental health, independent of other physiological, behavioral, and psychosocial risk factors [20–23].

### Other covariates

We selected additional measures of vulnerability as the other covariates, including age (≤ 17 years; 18–64 years), sex (male; female), education (college; General Educational Development [GED] or high school; none), employment status (employed; unemployed), census region (northeast; Midwest; west; south), perceived mental health status (excellent; very good; good; fair; poor), and need Activities of Daily Living (ADL) help (yes; no).

### Statistical analyses

We summarized the insurance coverage status by vulnerability groups as percentages (95% confidence intervals) (PROC SURVEYFREQ). We performed collinearity tests and used the criteria that the condition indices (CI) > 30 and 2 or more variance decomposition proportions (VDPs) > 0.5 to identify collinearity problems. Then we constructed three logistic regression models (PROC SURVEYLOGISTIC) to estimate the associations between insurance coverage status and vulnerability measures, comparing insured with uninsured or insured for part of the year, insured for part of the year only, and uninsured only, respectively.

We excluded observations with missing outcome (insurance coverage status) or exposures (race, family income, self-perceived health status). We treated missing values for the other covariates as a separate category for the multivariable logistic regressions to maximize sample size and hence analytic power. We performed data management and analyses using SAS 9.4 (SAS Institute, Cary, NC, USA), accounting for complex survey design, i.e., strata, cluster, and sampling weights. The designated statistical significance level was two-sided*p*-value < 0.05.

## Results

Of the 25,696 US civilian noninstitutionalized subjects that were under 65 years old in the analytical data set, 7208 (28.1%) subjects were classified into the white-highincome-good health group (Table 1). The majority of this group had private insurance (78.2, 95% CI: 76.6, 79.8%). On the other hand, 1022 (4.0%) subjects were classified into the minority-lowincome-bad health group, 54.5% of whom were covered by public insurance (95% CI: 50.6, 58.4%) (Table 1). Across the eight groups, the low-income minorities with bad health had the highest uninsured percentage (15.8, 95% CI: 12.6, 19.0%), followed by low-income minorities with good health (15.3, 95% CI: 13.7, 16.8), whereas the white-highincome-good health group had the lowest uninsurance rate (3.7, 95% CI: 3.0, 4.3).

**Table 1:** Vulnerability and health insurance coverage - 2017 US civilian noninstitutionalized population under 65 years old^1^

						***Insurance coverage***
***Vulnerability***	***N*** (%)	Public insurance	Private insurance	Private & public insurance	Partial insurance	Uninsured
Minority-low income-bad health	1022 (4.0)	54.5 (50.6, 58.4)	8.9 (7.1, 10.8)	5.6 (4.2, 7.0)	15.2 (12.6, 17.7)	15.8 (12.6, 19.0)
Minority-low income-good health	6783 (26.4)	46.5 (44.4, 48.6)	14.2 (12.8, 15.7)	5.9 (4.9, 6.9)	18.1 (16.6, 19.6)	15.3 (13.7, 16.8)
White-low income-bad health	511 (2.0)	53.4 (49.7, 57.0)	12.8 (9.7, 16.0)	8.5 (6.2, 10.7)	14.9 (11.5, 18.2)	10.4 (8.2, 12.7)
White-low income-good health	2408 (9.4)	39.0 (35.5, 42.5)	25.8 (22.5, 29.1)	8.2 (6.5, 9.9)	17.2 (15.2, 19.3)	9.8 (8.3, 11.3)
Minority-high income-bad health	541 (2.1)	17.4 (14.4, 20.4)	51.3 (46.9, 55.8)	8.6 (6.0, 11.1)	14.4 (11.0, 17.8)	8.3 (6.3, 10.3)
Minority-high income-good health	6742 (26.2)	9.9 (8.8, 11.0)	65.4 (63.4, 67.4)	4.8 (4.0, 5.6)	11.3 (10.3, 12.4)	8.6 (7.6, 9.7)
White-high income-bad health	481 (1.9)	15.6 (12.5, 18.7)	61.7 (57.9, 65.6)	6.0 (4.3, 7.7)	10.9 (8.8, 12.9)	5.9 (3.8, 7.9)
White-high income-good health	7208 (28.1)	4.9 (4.2, 5.6)7	78.2 (76.6, 79.8)	4.5 (3.7, 5.2)	8.7 (7.6, 9.8)	3.7 (3.0, 4.3)

^1^All values are proportions (95% confidence intervals) unless otherwise specified. Civilian noninstitutionalized population is defined as “All U.S. civilians not residing in institutional group quarters facilities such as correctional institutions, juvenile facilities, skilled nursing facilities, and other long-term care living arrangements”

(Reference: https://www.census.gov/glossary/#term_Civiliannoninstitutionalizedpopulation)

The observations with “Don’t know” were setting to missing and are not presented in this Table

^*^The subjects with missing ages were not included because we could not assume they were under 65 years old

^*^Family income comprised annual earnings from wages, salaries, bonuses, tips, commissions; business and farm gains and losses; unemployment and workman’s compensation; interest and dividends; alimony, child support, and other private cash transfers; private pensions, IRA withdrawals, social security, and veterans payments; supplemental security income and cash welfare payments from public assistance, aid to families with dependent children, and aid to dependent children; gains and losses from estates, trusts, partnerships, S corporations, rent, and royalties; and a small amount of “other” income. Family income excluded tax refunds and capital gains

There was a clear association between income level and health insurance coverage. Those with high income had much smaller uninsurance rate than those with low income: 3.7–8.6% vs. 9.8–15.8%, a difference of 6.1–7.2%. Likewise, those with high income had much smaller partial insurance rate than those with low income: 8.7–14.4% vs. 14.9–18.1%, a difference of 3.7–6.2%. Among the insured, those with high income were more likely to have private insurance (51.3–78.2% vs. 8.9–25.8%) whereas those with low income were more likely to have public insurance (39.0–54.5% vs. 4.9–17.4%) presumably due to the connection between low income and public insurance qualification.

At the same income level, minorities were more likely to be uninsured than Whites. For example, at the low-income level, 15.3–15.8% minorities were uninsured depending on their health status whereas 9.8–10.4% Whites were uninsured, a difference of about five percentage points. At the high-income level, 8.3–8.6% minorities were uninsured depending on their health status whereas 3.7–5.9% Whites were uninsured, a difference of about four percentage points. Likewise, minorities were more likely to be insured for part of the year than Whites. For example, at the low-income level, 15.2–18.1% minorities were insured for part of the year depending on their health status whereas 14.9–17.2% Whites were insured for part of the year. At the high-income level, 11.3–14.4% minorities were insured for part of the year depending on their health status whereas 8.7–10.9% Whites were insured for part of the year.

Table 2 showed the results of collinearity tests. The collinearity problems were identified when condition indices are (CI) > 30 and 2 or more variance decomposition proportions (VDPs) are > 0.5. The results indicated that the original models only had collinearity problem when age was included. After dropping age, our models did not have collinearity problems (CI < 30).

**Table 2:** Collinearity Diagnostics

	Model 1	Model 2	Model 3	Model 1 (without age)	Model 2 (without age)	Model 3 (without age)
Condition Indices	30.4	30.2	33.8	13.4	13.6	13.0
Variance Decomposition Proportion						
Intercept	0.9716	0.9735	0.9688	0.9883	0.9897	0.9866
group	0.0062	0.0056	0.0094	0.2239	0.2374	0.2067
age_3c	0.8713	0.8601	0.91	–	–	–
female	0.0056	0.0052	0.0039	0.0435	0.0459	0.0439
edu	0.2839	0.2939	0.2273	0.0044	0.0041	0.0034
employed	0.1724	0.1798	0.1427	0.0558	0.0596	0.0517
region	0.0518	0.0531	0.0355	0.4996	0.475	0.5258
mentalhealth53	0.0351	0.042	0.0283	0.2761	0.2975	0.2684
adlhelp31	0.0045	0.0062	0.0003	0.0003	0	0.0001

Collinearity problems indicated when: Condition Indices (CI) > 30 AND 2 or more Variance Decomposition Proportions (VDPs) > 0.5. The original models have collinearity problems that is cause by age. After dropping age, our models did not have collinearity problems (CI < 30)

The results of logistic regression models are shown in Table 3. Compared with the high-income Whites with good health (the least vulnerable group), the other groups had lower odds of becoming insured, after adjusting for sex, education, employment status, census region, perceived mental health status, and ADL help (Table 3). Low-income minorities with bad health had 68% less odds of being insured instead of uninsured or insured for part of the year than high-income Whites with good health (odds ratio [OR]: 0.32; 95% CI: 0.24, 0.42) (Table 3, Model 1). Similarly, compared with high-income Whites with good health, the odds of being insured among low-income minorities with good health, high-income minorities with bad health, high-income minorities with good health, low-income Whites with bad health, and low-income Whites with good health were 74% (OR: 0.26; 95% CI: 0.22, 0.32), 45% (OR: 0.55; 95% CI: 0.40, 0.74), 42% (OR: 0.58; 95% CI: 0.49, 0.68), 54% (OR: 0.46; 95% CI: 0.33, 0.64), and 63% (OR: 0.37; 95% CI: 0.30, 0.45), respectively (Table 3, Model 1). Additional analyses using the insured for part of the year and uninsured levels as reference groups yielded analogous results (Table 3, Model 2 and 3). In terms of results related to covariates, we found compared to unemployed group, employed had lower odds of having insurance (OR: 0.71; 95% CI: 0.64, 0.80) (Table 3, Model 1).

**Table 3:** Logistic regressions of vulnerability, sociodemographic characteristics, and insurance status - 2017 U.S. Civilian noninstitutionalized population under 65 years old

Independent Variables	Model 1 (1 = Insured 0 = Uninsured or partially insured)	Model 2 (1 = Insured 0 = Partially insured)	Model 3 (1 = Insured 0 = Uninsured)
Number of Observations Used	25,696	23,135	22,380
Intercept	3.68 (2.43, 5.57)^c^	5.64 (3.38, 9.41)^c^	14.21 (7.55, 26.75)^c^
Vulnerability			
Minority-low income-bad health	0.32 (0.24, 0.42)^c^	0.43 (0.30, 0.61)^c^	0.19 (0.13, 0.27)^c^
Minority-low income-good health	0.26 (0.22, 0.32)^c^	0.34 (0.28, 0.43)^c^	0.16 (0.13, 0.21)^c^
White-low income-bad health	0.55 (0.40, 0.74)^c^	0.55 (0.37, 0.80)^b^	0.52 (0.36, 0.77)^c^
White-low income-good health	0.58 (0.49, 0.68)^c^	0.69 (0.57, 0.84)^c^	0.42 (0.33, 0.53)^c^
Minority-high income-bad health	0.46 (0.33, 0.64)^c^	0.52 (0.35, 0.79)^b^	0.34 (0.22, 0.53)^c^
Minority-high income-good health	0.37 (0.30, 0.45)^c^	0.40 (0.32, 0.51)^c^	0.29 (0.22, 0.39)^c^
White-high income-bad health	0.79 (0.56, 1.10)	0.83 (0.56, 1.22)	0.69 (0.42, 1.16)
White-high income-good health	Reference group	Reference group	Reference group
Sex			
Male	0.79 (0.74, 0.85)^c^	0.90 (0.82, 0.97)^a^	0.65 (0.59, 0.72)^c^
Female	Reference group	Reference group	Reference group
Education			
GED/high school	1.36 (1.22, 1.52)^c^	1.12 (0.97, 1.29)	1.62 (1.40, 1.87)^c^
College	2.98 (2.55, 3.49)^c^	1.99 (1.63, 2.42)^c^	5.11 (3.93, 6.64)^c^
None	Reference group	Reference group	Reference group
Employment status			
Employed	0.71 (0.64, 0.80)^c^	0.75 (0.66, 0.86)^c^	0.64 (0.55, 0.74)^c^
Unemployed	Reference group	Reference group	Reference group
Census region			
Northeast	1.90 (1.58, 2.29)^c^	1.59 (1.28, 1.96)^c^	2.61 (1.95, 3.50)^c^
Midwest	1.43 (1.20, 1.69)^c^	1.25 (1.04, 1.50)^a^	1.77 (1.36, 2.30)^c^
West	1.40 (1.21, 1.63)^c^	1.18 (0.99, 1.40)	1.88 (1.54, 2.30)^c^
South	Reference group	Reference group	Reference group
Perceived mental health status			
Excellent	1.21 (0.83, 1.77)	1.57 (1.01, 2.45)^a^	0.76 (0.41, 1.41)
Very good	1.29 (0.88, 1.88)	1.56 (1.00, 2.43)	0.90 (0.50, 1.63)
Good	1.13 (0.77, 1.66)	1.45 (0.93, 2.28)	0.73 (0.40, 1.32)
Fair	1.35 (0.91, 1.98)	1.55 (0.99, 2.42)	1.03 (0.54, 1.97)
Poor	Reference group	Reference group	Reference group
Need ADL help			
Yes	4.13 (2.45, 6.95)^c^	4.03 (2.01, 8.08)^c^	4.19 (2.00, 8.81)^c^
No	Reference group	Reference group	Reference group

All values are odds ratios (95% confidence intervals) unless otherwise specified. ^a^ *P* < 0.05, ^b^ *P* < 0.01, ^c^ *P* < 0.001

## Discussion

This study used the vulnerability framework and assessed the combined and individual effect of race and income on health insurance coverage in the US. Results of the study showed that while income was a significant predictor of health insurance coverage (a difference of 6.1–7.2% between high- and low-income Americans), race/ethnicity was independently associated with lack of insurance. At the same income level, minorities were significantly more likely to be uninsured than Whites ranging from four percentage-point difference at the high-income level to five percentage-point difference at the low-income level. Moreover, minorities were more likely to be insured for part of the year than Whites. There were 31.0–33.4% low-income minorities who were either uninsured or insured for part of the year during the year compared to 25.3–27.0% for low-income whites, a difference of about six percentage point. There were 19.9–22.7% high-income minorities who were either uninsured or insured for part of the year during the year compared to 12.4–16.8% for high-income Whites, also a difference of about six percentage point. The combined effect of income and race on insurance coverage was devastating as low-income minorities with bad health had 68% less odds of being insured instead of uninsured or insured for part of the year than high-income Whites with good health. It is also noteworthy that minorities were disproportionately over-represented in the low-income or bad health groups so that any adverse association between income, bad health, and insurance status would more adversely affect minorities than Whites.

Our findings were consistent with previous studies. Buchmueller and Levy evaluated the ACA’s impact on racial and ethnic disparities in health insurance coverage and access to care, and found a large number of adults remain uninsured, and the uninsurance rate among blacks and Hispanics was substantially higher than the rate among whites [24]. Another study assessed the insurance patterns and instability from 2006 to 2016, and found despite the post-ACA instability reduction, over 25% of the U.S. population continued to have insurance gaps over a two-year period; and found disparities continued to exist between income groups, race/ethnicities, and regions [25]. Compared with the above evidence, our results added further empirical evidence on the combined effect of race and income on health insurance coverage in the US, and the uniqueness of our study was looking at the influence of multiple vulnerability traits together rather than separately as other studies did.

These findings have significant policy implications. To expand insurance coverage and eliminate racial disparities as called for by Healthy People 2030, concerted and targeted efforts are needed to both improve economic status and address disparities towards racial/ethnic minorities. To maximize limited resources, policymakers should target insurance coverage for the most vulnerable subpopulations, i.e., those with a convergence of predisposing, enabling, and need attributes of risk. The findings placed quantitative content to the theoretical framework of the vulnerability mode. As shown in this study, these are individuals who have low income and are racial/ethnic minorities. Significant progress at reducing disparities in care and quality across racial and ethnic groups is unlikely to be fulfilled without significantly expanding insurance coverage for this most vulnerable group.

There were a number of limitations with this study. First, the cross-sectional nature of the dataset made it impossible to establish the causal connection between individual socioeconomic characteristics and insurance coverage. Only longitudinal or cohort study could overcome this limitation. Second, the limitation of unavailability of variables in health behavior in MEPS prevented us from studying a broad array of vulnerable characteristics particularly measures of behavioral risks such as smoking, alcohol consumption, and drug abuse, all of which likely to contribute to vulnerability. Third, due to limited sample size and therefore lack of power, the current study did not study racial/ethnic populations separately nor further dissected the vulnerability traits and their interactions. For example, it could well be that Asians performed better in healthcare access due to their general higher income and education status and by including them with other minorities, the overall disparities might be reduced as a result.

As discussed the current study was limited by the availability of data. Future research could more fully incorporate the vulnerability framework to address the interactions of multiple risk (vulnerability) factors on health and healthcare and identify vulnerability traits most likely to exacerbate poor health and healthcare. Further research is also needed to elucidate the trajectories or pathways that these vulnerability traits interact to affect health and healthcare and their precursors. Future research could also study different racial/ethnic populations separately for differences within racial/ethnic populations are likely and analyzing them together could moderate the actual size of the disparities.

Despite these limitations, our study contributes to the literature on predictors of insurance coverage and has practical implications. For example, having insurance coverage is critical to accessing the health system and is particularly important during pandemics like COVID-19. Having health insurance and the resulting usual source of care is paramount for getting timely and coordinated care. People without health insurance and a usual place to go when they need medical care will likely face unique barriers accessing COVID-19 testing and treatment services. They may not know where to go to get tested if they think they have been exposed to the virus and may forego testing or care out of fear of having to pay out-of-pocket for the test. Moreover, racial/ethnic minority groups are more likely to disproportionately experience adverse health outcomes that are worse and more acute than non-minority groups, underscoring the need for timely access to testing, treatment, and follow-up care. The Emergency Medical Treatment and Labor Act requires hospitals to screen and stabilize patients with emergent conditions, however, they are not required to provide treatment for non-emergent conditions. Access barriers are magnified in times of crisis like COVID-19 and are unlikely to be fundamentally addressed without ultimately expanding health insurance coverage to all Americans.

## Conclusion

The current study found the combined effect of income and race on insurance coverage was devastating as low-income minorities with bad health had 68% less odds of being insured than high-income Whites with good health. Results of the study could assist policymakers in targeting limited resources on subpopulations likely most in need of assistance for insurance coverage. Policymakers should target insurance coverage for the most vulnerable subpopulation, i.e., those who have low income and poor health as well as are racial/ethnic minorities.

## Data Availability

The data that support the findings of this study are available from Medical Expenditure Panel Survey, https://meps.ahrq.gov/mepsweb/.
